# Mapping the structure of biomarkers in autism spectrum disorder: a review of the most influential studies

**DOI:** 10.3389/fnins.2024.1514678

**Published:** 2024-12-13

**Authors:** Fang Jin, Zhidan Wang

**Affiliations:** School of Education Science, Jiangsu Normal University, Xuzhou, China

**Keywords:** autism biomarkers, bibliometric analysis, genetics, brain, metabolomics, oxidative stress, mitochondrial dysfunction

## Abstract

**Background:**

Autism spectrum disorder is a distinctive developmental condition which is caused by an interaction between genetic vulnerability and environmental factors. Biomarkers play a crucial role in understanding disease characteristics for diagnosis, prognosis, and treatment. This study employs bibliometric analysis to identify and review the 100 top-cited articles’ characteristics, current research hotspots and future directions of autism biomarkers.

**Methods:**

A comprehensive search of autism biomarkers studies was retrieved from the Web of Science Core Collection database with a combined keyword search strategy. A comprehensive analysis of the top 100 articles was conducted with CiteSpace, VOSviewer, and Excel, including citations, countries, authors, and keywords.

**Results:**

The top 100 cited studies were published between 1988 and 2021, with the United States led in productivity. Core biomarkers such as genetics, children, oxidative stress, and mitochondrial dysfunction are well-established. Potential trends for future research may include brain studies, metabolomics, and associations with other psychiatric disorders.

**Conclusion:**

This pioneering bibliometric analysis provides a comprehensive compilation of the 100 most-cited studies on autism, which not only offers a valuable resource for doctors, and researchers but shedding insights into current shortcomings and future endeavors. Future research should prioritize the application of emerging technologies for biomarkers, longitudinal study of biomarkers, and specificity of autism biomarkers to advance the precision of ASD diagnosis and treatment.

## 1 Introduction

Autism spectrum disorder (ASD) is a lifelong neurodevelopmental disorder defined by difficulties with social interaction and communication, as well as patterns of restricted, repetitive behaviors. According to the Centers for Disease Control and Prevention (CDC), the prevalence of ASD has risen from 1 in 150 by 2000 to 1 in 36 by 2020, indicating a notable increase over recent decades. ASD can profoundly effects on cognitive abilities, adaptive skills, and psychological functioning throughout an individual’s life (Cai et al., 2019; Leader et al., 2021).

Given the clinical diversity of ASD, researchers and clinicians have focused on identifying markers to improve diagnosis, classification, and treatment prediction. Markers—indicators of disease states or treatment responses—are valuable tools in ASD research (Lord et al., 2018). Current studies have made considerable strides in identifying behavioral, psychological, and biological markers, each providing unique insights into ASD (Baron-Cohen et al., 1996; Tager-Flusberg, 1999; Bishop et al., 2017; Micai et al., 2020; Shen et al., 2020; Hiremath et al., 2021).

Behavioral markers are essential for diagnosing ASD, as they are visible in daily life and guide intervention strategies. Research has shown that social interaction and communication challenges, along with repetitive behaviors, and restricted interests, are key behavioral markers of ASD (Lord et al., 2000; Tager-Flusberg et al., 2005; Georgiades et al., 2013). Many individuals with ASD struggle to make eye contact or interpret social cues such as facial expressions, gestures, and tone of voice. They also exhibit significant communication deficits, including delayed language development and difficulties with nonverbal communication (gestures and body language; Mundy et al., 1986; Tager-Flusberg et al., 2005). Additionally, repetitive behaviors in autism are characterized by stereotyped movements with objects, a strong and persistent interest in specific topics or objects, and high levels of focus in activities (Richler et al., 2010; Leekam et al., 2011). Based on these markers, diagnostic tools such as the Modified Checklist for Autism in Toddlers (M-CHAT), have been developed, alongside interventions like Applied Behavior Analysis (ABA), Early Intensive Behavioral Intervention (EIBI), and social skills training, which helps improve communication and social engagement (Robins et al., 2001; Reichow et al., 2012; Smith and Iadarola, 2015).

Psychological markers further highlight ASD’s impact, revealing distinct patterns in language comprehension, theory of mind, and executive function that shape the autistic experience. Many individuals with autism show delayed language development, often struggling with complex sentences, metaphors, and implied meanings, making communication more challenging (Tager-Flusberg, 2000; Eigsti et al., 2011). A key psychological feature of ASD is a deficit in theory of mind—the ability to comprehend others’ thoughts, beliefs, and intentions—which can hinder social interactions (Happé, 1995; Tager-Flusberg, 2007; Cerullo et al., 2021). Additionally, those with ASD show difficulties in executive function, such as working memory, cognitive flexibility, and planning abilities. Studies have documented reduced short-term memory capacity in individuals with ASD, including challenges in digit span and visuospatial tasks (Habib et al., 2019). They also have significant trouble switching tasks and adapting to new environments, as assessments using the Stroop task and other task-switching tests have demonstrated slower response times and higher error rates in this population (Stoet and López, 2011; Leung and Zakzanis, 2014; Demetriou et al., 2018).

Biomarkers provide a unique and objective foundation for understanding ASD, focusing on measurable biological indicators that could clarify its causes, improve diagnostic accuracy, and support treatment development (Ecker et al., 2013; Chen et al., 2024). Unlike behavioral and psychological markers, biological markers can provide consistent, quantifiable measurements, which help reduce diagnostic variability and enhance the consistency of ASD assessments across different clinical settings. This consistency is key to establishing standardized diagnostic criteria internationally. Biological markers, such as genetic mutations, specific metabolites, and brain imaging features, can often be identified in early childhood or even prenatally, enhancing the potential for earlier diagnosis and intervention. For instance, non-invasive testing of biological samples, such as blood, urine, or saliva, may help identify high-risk individuals before behavioral symptoms emerge, streamlining the diagnostic process and reducing costs associated with extended behavioral assessments.

In recent years, autism biomarker research has rapidly expanded, driven by advancements in neuroimaging, genetics, and biochemistry (Frazier et al., 2014; Charman et al., 2017). Genetic studies have identified specific mutations and variations that are closely associated with ASD, providing insights into its genetic foundations. Mutations in genes such as CHD8, SHANK3, and SCN2A have shown strong links to ASD (Durand et al., 2007; Schaaf and Zoghbi, 2011; Sanders et al., 2012; Bernier et al., 2014). Additionally, copy number variations (CNVs) such as the 15q11-13 duplication and 16p11.2 deletion, are strongly correlated with ASD (Marshall et al., 2008; Weiss et al., 2008; Kumar and Christian, 2009; Pinto et al., 2010; Schaaf and Zoghbi, 2011). Large-scale genome-wide association studies (GWAS) and whole-genome sequencing continue to unravel ASD’s complex genetic landscape, revealing rare, high-impact genetic variants that appear to influence autism development significantly, with certain genes like DYRK1A and ADNP linked to more severe ASD phenotypes (De Rubeis et al., 2014).

In neuroimaging, MRI and fMRI are widely used to examine brain structure and function in ASD (Ecker et al., 2010; Philip et al., 2012). Findings reveal distinct structural and functional connectivity abnormalities, particularly in brain areas like the frontal and temporal cortex, amygdala, and hippocampus—regions associated with social behavior and emotional regulation (Stigler et al., 2011; Haar et al., 2016). Diffusion tensor imaging (DTI) further indicates abnormalities in the integrity and connectivity of white matter tracts, potentially underlying challenges in information processing and cognitive functions in ASD (Brito et al., 2009; Travers et al., 2012).

Biochemical markers, such as neurotransmitter levels, metabolites, and immune responses, also offer valuable insights. Individuals with autism often show atypical levels of metabolites in serum, urine, and cerebrospinal fluid. For instance, shifts in oxidative stress markers, inflammatory factors, and amino acids are thought to relate to ASD’s pathology. Neurotransmitter research shows that imbalances in glutamate and *γ*-aminobutyric acid (GABA) may significantly affect ASD’s neural mechanisms (Pizzarelli and Cherubini, 2011). Additionally, abnormal immune responses, including the presence of autoantibodies and altered cytokine levels, suggest an immunological dimension to autism (Vargas et al., 2005; Masi et al., 2017).

However, ASD biomarker research faces several challenges, including fragmented efforts, difficulty in tracking emerging research trends, and the complexity of integrating diverse findings. Considering the expanding output and dynamic evolution of autism biomarkers research, it is increasingly essential to use quantitative methods to assess and analyze the existing body of work. Bibliometrics, a statistical analysis method, is key in identifying influential papers, emerging trends, and research hotspots through co-word and co-citation analyses within specific fields (Van Raan, 2014). Citation analysis, a central component of bibliometric analyses, is a valuable tool for assessing the impact of articles and tracking the evolution of a research domain (Garfield, 1972). By examining the most cited studies, particularly the top 100 cited studies, are often seminal works that have significantly influenced the field. Highlighting these contributions offers a clear picture of the key developments in autism biomarker research. However, there is a notable absence of focused studies on autism biomarkers.

Therefore, this study aims to bridge this gap by conducting a comprehensive bibliometric analysis of autism biomarkers research, focusing on identifying and characterizing the 100 most cited studies in this field. The analysis encompasses a thorough investigation into the bibliometric characteristics of these articles. It will provide an interdisciplinary perspective on the characteristics of autism biomarkers research, offering a detailed exploration of currently highly cited articles. Furthermore, the thorough examination of keywords will help identify emerging trends and research hotspots in autism biomarkers, shedding light on the future directions of the field. These findings will serve as a valuable resource for a wide range of professionals, including epidemiologists, pediatricians, rehabilitation therapists, and caregivers, all of whom are working to advance their understanding and practice in relation to autism spectrum disorder.

## 2 Materials and methods

### 2.1 Search strategy

On June 8th, 2024, a comprehensive search was conducted in the Web of Science Core Collection (WoSCC) database, hosted by Clarivate Analytics.^1^ The Web of Science was chosen for its multidisciplinary scope, offering access to both current and retrospective data dating back to 1900.

Despite the Diagnostic and Statistical Manual of Mental Disorders (Fifth Edition; DSM-5) broadening the definition of “autism spectrum disorder” (ASD) and discontinuing subdivisions like “pervasive developmental disorder not otherwise specified,” “autistic disorder,” and “Asperger syndrome,” these terms remain prevalent in clinical practice and research (Kim et al., 2014). For this study, the search strategy was: [TS = (autistic OR autism OR ASD OR Asperger OR Heller’s syndrome OR pervasive developmental disorder OR dementia infantilis OR disintegrative disorder OR Kanner’s syndrome)] AND [TS = (biomarker* OR marker*)].

Articles were retrieved based on total citation count in descending order, with more recent articles prioritized in cases of identical citation counts.

### 2.2 Data exaction

Articles included in the analysis were sourced from indexed journals and specifically focused on autism biomarkers. Only original research articles and reviews were considered, while editorials, letters, conference proceedings, meeting reports, books, book chapters, and documents of undefined types were excluded.

To ensure relevance to this study’s objectives, the results were meticulously reviewed. Subsequently, the top 100 most cited papers were selected for detailed analysis.

Data extracted from each paper included the title, total citation count, authorship, institution, country, language, publication year, journal title, document type, journal impact factor, and Web of Science subject category. Journal impact factors (IF) were determined using the 2022 Journal Citation Reports.^2^

### 2.3 Statistical analysis

Data analysis and visualization were carried out using MS Excel (version 16.0), SPSS (version 26), CiteSpace (version 6.2.4), and VOSviewer (version 1.6.15).

MS Excel was utilized for quantitative data analysis and basic visualizations to display trends and statistics. SPSS was employed to analyze the relationships, such as the correlation between study count and journal impact factors.CiteSapce, a scientometrics tool, visualizes the structure and trends of scientific knowledge, generating “knowledge maps” to reflect the field’s progress (Xiao et al., 2023). It was used to analyze keyword timelines, burst keywords, and clusters of countries, institutions, authors, and keywords. VOSviewer, another bibliometric tool, creates and visualizes networks based on citations, co-citation, bibliographic coupling, or co-authorship (Xiao et al., 2023). Nodes represent elements like countries, institutions, and keywords, with node size indicating publication volume and link width showing collaboration strength (Zhang et al., 2022). This study utilized VOSviewer to analyze collaboration networks and keyword overlays.

## 3 Results

### 3.1 Basic characteristics of the 100 top-cited studies

**Figure 1:**
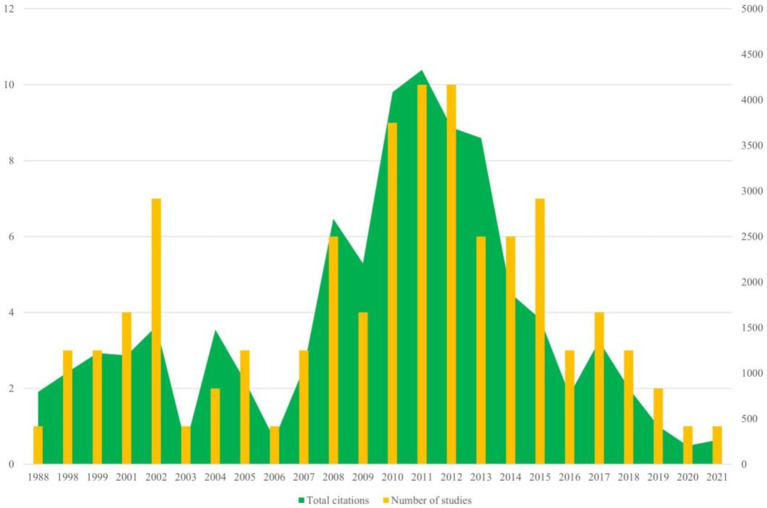
Annual number of publications of autism biomarkers research.

The 100 top-cited articles are listed in Supplementary Table 1. The 100 top-cited studies on autism biomarkers were all published in English, spanning from 1988 (by Courchesne et al.) to 2021 (by Maynard et al.). Nearly 50% (53 articles) of these studies published since the year 2010. The most productive years were 2011 and 2012, each contributing 10 articles. Among these, 2011 stood out not only for the highest total citation count but also for having the highest average citation count per study, with an average of 432.8 citations per article (see Figure 1).

These articles were cited ranging from 180 to 2,088 times. The article with the highest number of citations “Identification of risk loci with shared effects on five major psychiatric disorders: A genome-wide analysis” written by Smoller was published in the Lancet in 2013 (Table 1).

**Table 1:** The 10 top-cited articles in autism biomarkers, ordered by number of citations.

Rank	Title	First author	Journal	Publication year	Total citations	Citation per year
1	Identification of risk loci with shared effects on five major psychiatric disorders: A genome-wide analysis	Smoller, J. W.	Lancet	2013	2088	189.82
2	Consensus statement: Chromosomal microarray is a first-tier clinical diagnostic test for individuals with developmental disabilities or congenital anomalies	Miller, D. T.	American Journal of Human Genetics	2010	1883	134.50
3	Transcriptomic analysis of autistic brain reveals convergent molecular pathology	Voineagu, I.	Nature	2011	1,316	101.23
4	Autism genome-wide copy number variation reveals ubiquitin and neuronal genes	Glessner, J. T.	Nature	2009	1,020	68.00
5	The genetics of autism	Muhle, R.	Pediatrics	2004	812	40.60
6	Hypoplasia of cerebellar vermal lobule VI and VII in autism	Courchesne, E.	New England Journal of Medicine	1988	795	22.08
7	Using Support Vector Machine to identify imaging biomarkers of neurological and psychiatric disease: A critical review	Orrù, G.	Neuroscience and Biobehavioral Reviews	2012	717	59.75
8	Metabolic biomarkers of increased oxidative stress and impaired methylation capacity in children with autism	James, S. J.	American Journal of Clinical Nutrition	2004	663	33.15
9	Gastrointestinal flora and gastrointestinal status in children with autism-comparisons to typical children and correlation with autism severity	Adams, J. B.	BMC Gastroenterology	2011	652	50.15
10	Oxidative stress in psychiatric disorders: evidence base and therapeutic implications	Ng, F.	International Journal of Neuropsychopharmacology	2008	625	39.06

Meanwhile, the 100 top-cited studies on autism biomarkers were published in 61 different journals, with the most frequent being “Molecular Psychiatry” (*n* = 9) and “American Journal of Human Genetics” (*n* = 9). Table 2 displays the journals hosting the 100 top-cited studies on autism biomarkers along with their associated impact factors. The journal impact factors of the 100 top-cited studies on autism biomarkers ranged from 2.3 to 168.9. However, no significant relationship was observed between the number of studies published in a journal and its impact factor.

**Table 2:** Top Journals of the 100 top-cited studies.

Journal	Number of studies	Citations	IF
Molecular Psychiatry	9	4,690	11
American Journal of Human Genetics	9	2,741	9.8
New England Journal of Medicine	3	768	158.5
Nature	3	659	64.8
Proceedings of the National Academy of Sciences of the United States of America	3	1,175	11.1
Biological Psychiatry	3	970	10.6
Translational Psychiatry	3	1,173	6.8
Human Molecular Genetics	3	1,225	3.5

### 3.2 Contributions of countries and organizations

**Figure 2:**
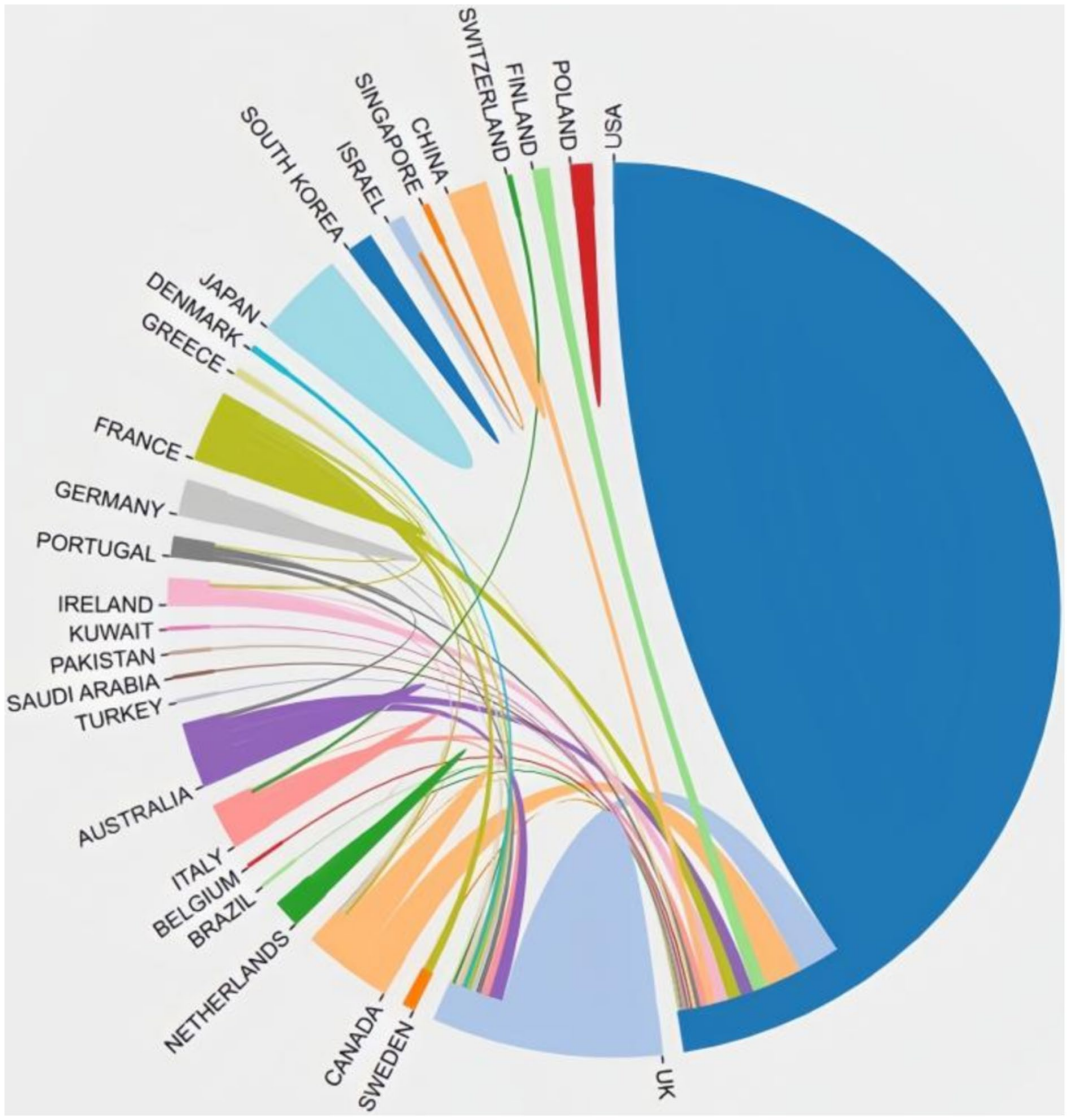
Cooperation network between countries.

In total, 29 countries contribute to the 100 top-cited publications. The three most productive countries were the United States (*n* = 69), England (*n* = 15), and Australia (*n* = 9). The US collaborated extensively with England, Canada, Netherlands, Finland, Australia, and France, highlighting their significant involvement in this field. Figure 2 illustrates the co-authorship map of these countries.

A total of 36 institutes contributed more than two publications each (see Table 3). The most prolific institutes were the Harvard University and the University of California, Los Angeles. Close behind was the University of Chicago and Columbia University in the city of New York. Notably, most of the top-cited institutions belong to the United States, demonstrating the authority and significance of the country in the field.

**Table 3:** Institutes that published at least five in the top 100 most-cited publications.

Institute	Number of studies	Citations
Harvard University	9	4,495
University of California, Los Angeles	9	4,031
The University of Chicago	7	3,675
Columbia University in the City of New York	7	1794
Massachusetts General Hospital	6	2,865
University of California, San Diego	6	2,387
The University of Utah	5	4,112
University of Pennsylvania	5	4,097
Johns Hopkins University	5	3,161
Vanderbilt University	5	2,342
Stanford University	5	2096
University of Oxford	5	1953
University of Arkansas for Medical Sciences	5	1917
University of Cambridge	5	1,517

### 3.3 Most contributing authors

The 100 studies involved 979 authors, among whom 17 authors contributed to at least 3 articles. Seven articles were authored individually.

**Figure 3:**
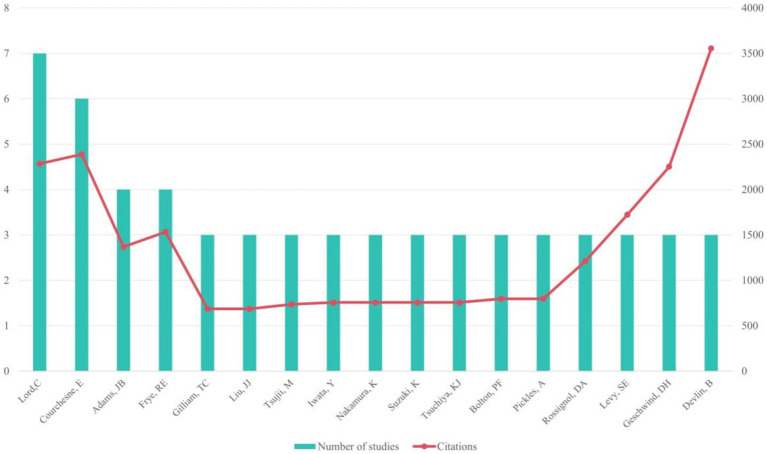
Authors who participated in at least two articles in the top 100 most-cited publications.

Among the most productive authors, Lord, C. led with 7 papers, followed by Courchesne, E. with 6 papers, and Adams, J.B. and Frye, R.E. with 4 papers each. Courchesne, E. accrued the highest total citations (*n* = 2,387), while Devlin, B.’s article had the highest average number of citations (*n* = 1184.67; see Figure 3). Meanwhile, Rossignol, D.A. led with the most T100 articles (*n* = 4) as the first author.

**Figure 4:**
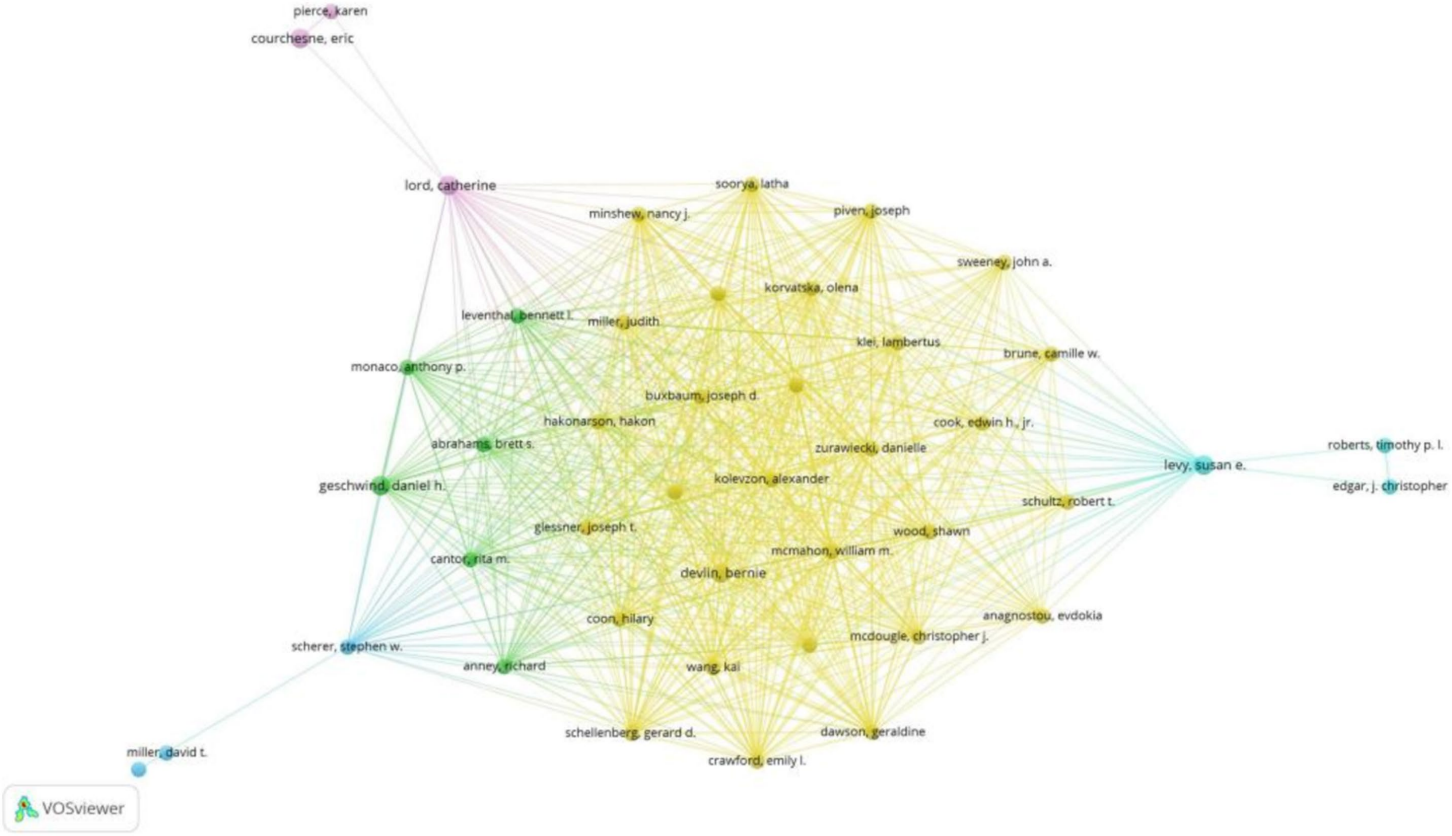
Co-authorship overlay visualization map of authors.

Figure 4 depicts the author collaboration network, highlighting Devlin, B. with the highest Total Structural Loss (TLS = 257) and collaborations with 233 authors. Notably, Devlin, B. closely collaborated with Sutcliffe, J.S., Wassink, T.H., Glessner, J.T., Coon, H., and Kolevzon, A.

### 3.4 Analysis of keywords

**Figure 5:**
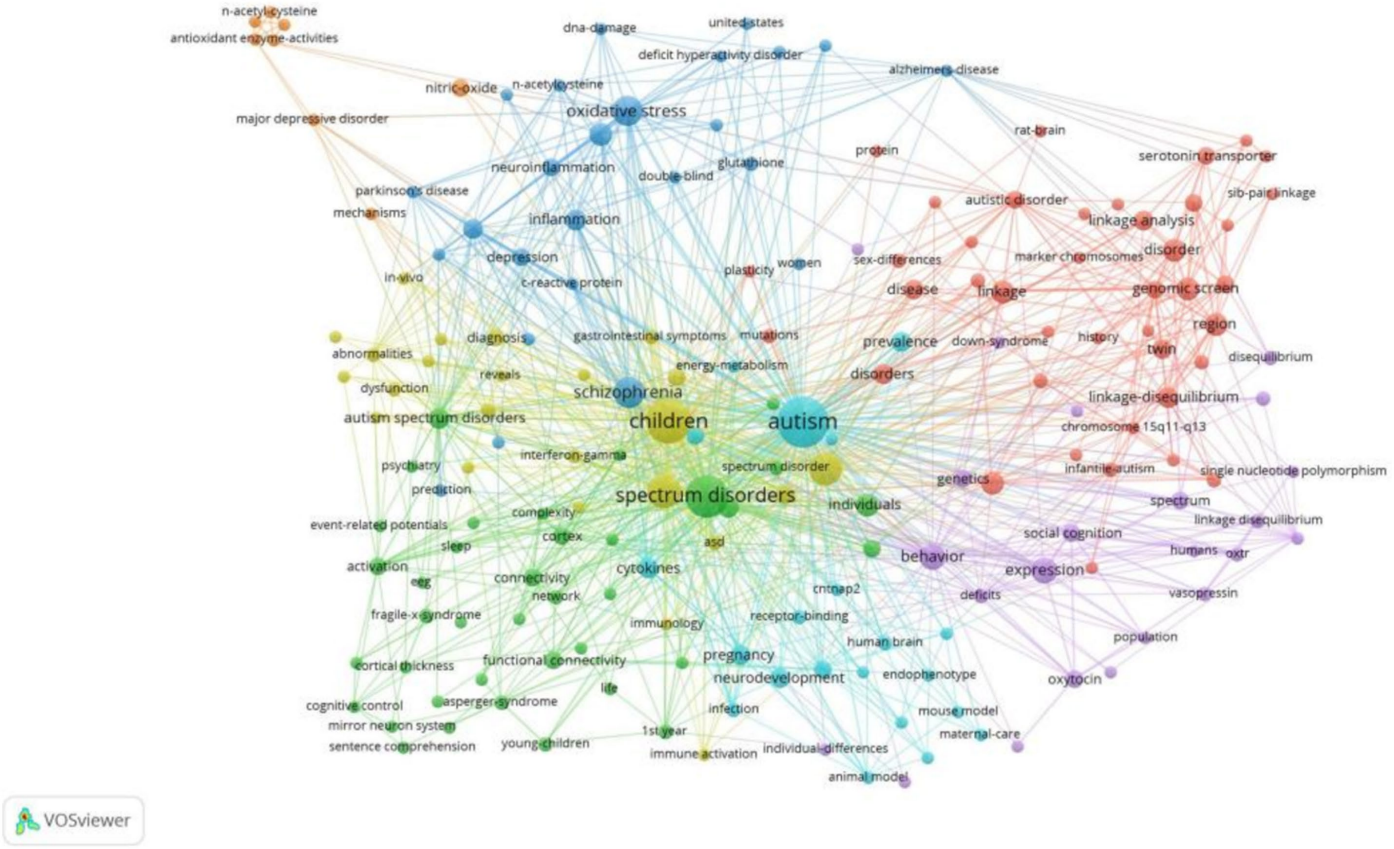
Map of keywords clustering in autism biomarkers research.

Keywords serve as concise summaries of topics discussed in an article, with high-frequency keywords indicating popular topics in a research field. The most frequent keywords were “children” (*n* = 27), “brain” (*n* = 16), “association” (*n* = 15), “schizophrenia” (*n* = 13), and “oxidative stress” (*n* = 12; see Figure 5).

**Figure 6:**
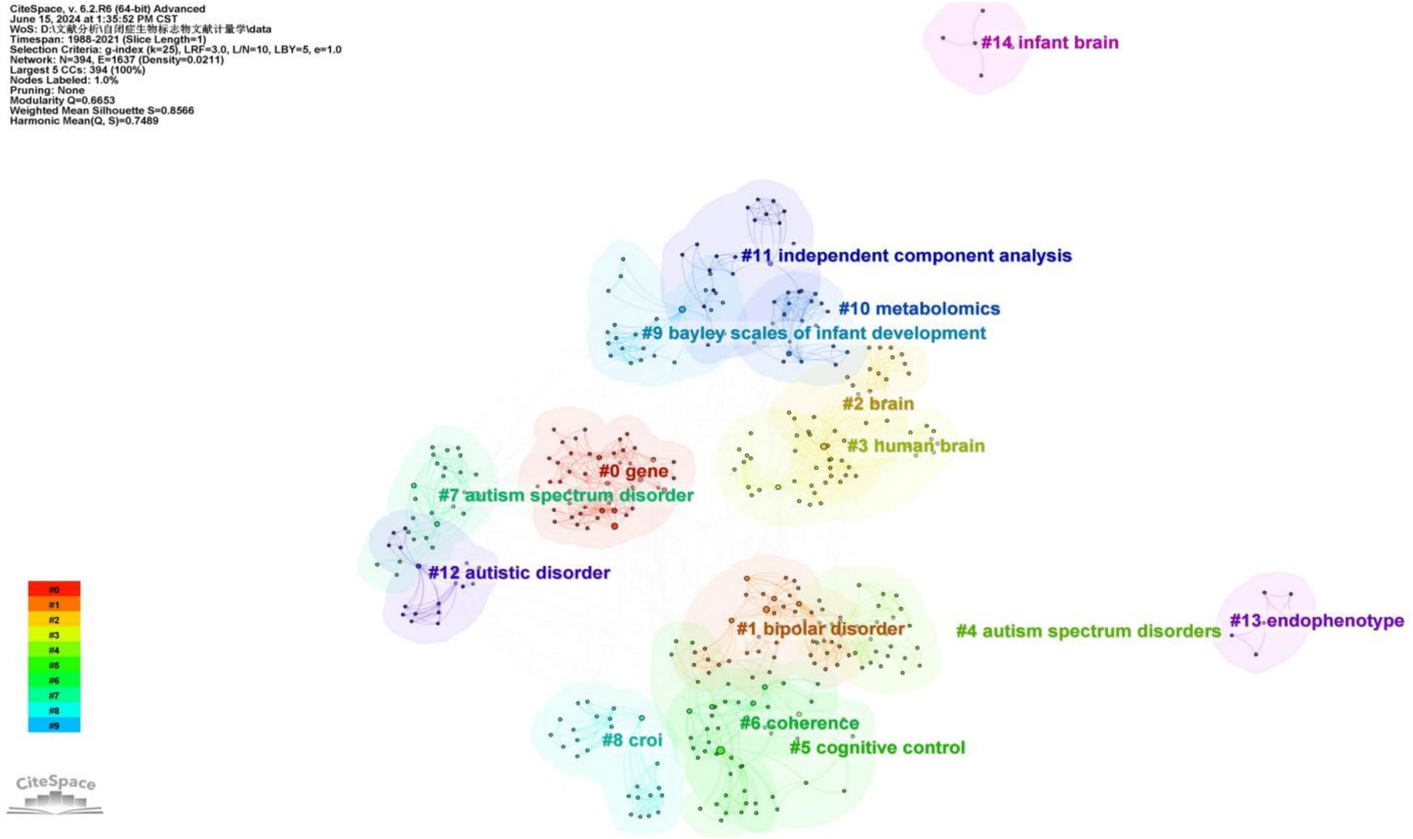
The cluster map of keywords.

A cluster analysis of co-occurring keywords was conducted using CiteSpace resulting in the classification of keywords into eleven distinct clusters based on their correlations. Figure 6 displays the top five largest clusters after removing non-exact clustering were “gene,” “bipolar disorder” “brain,” “cognitive control,” and “coherence”.

**Figure 7:**
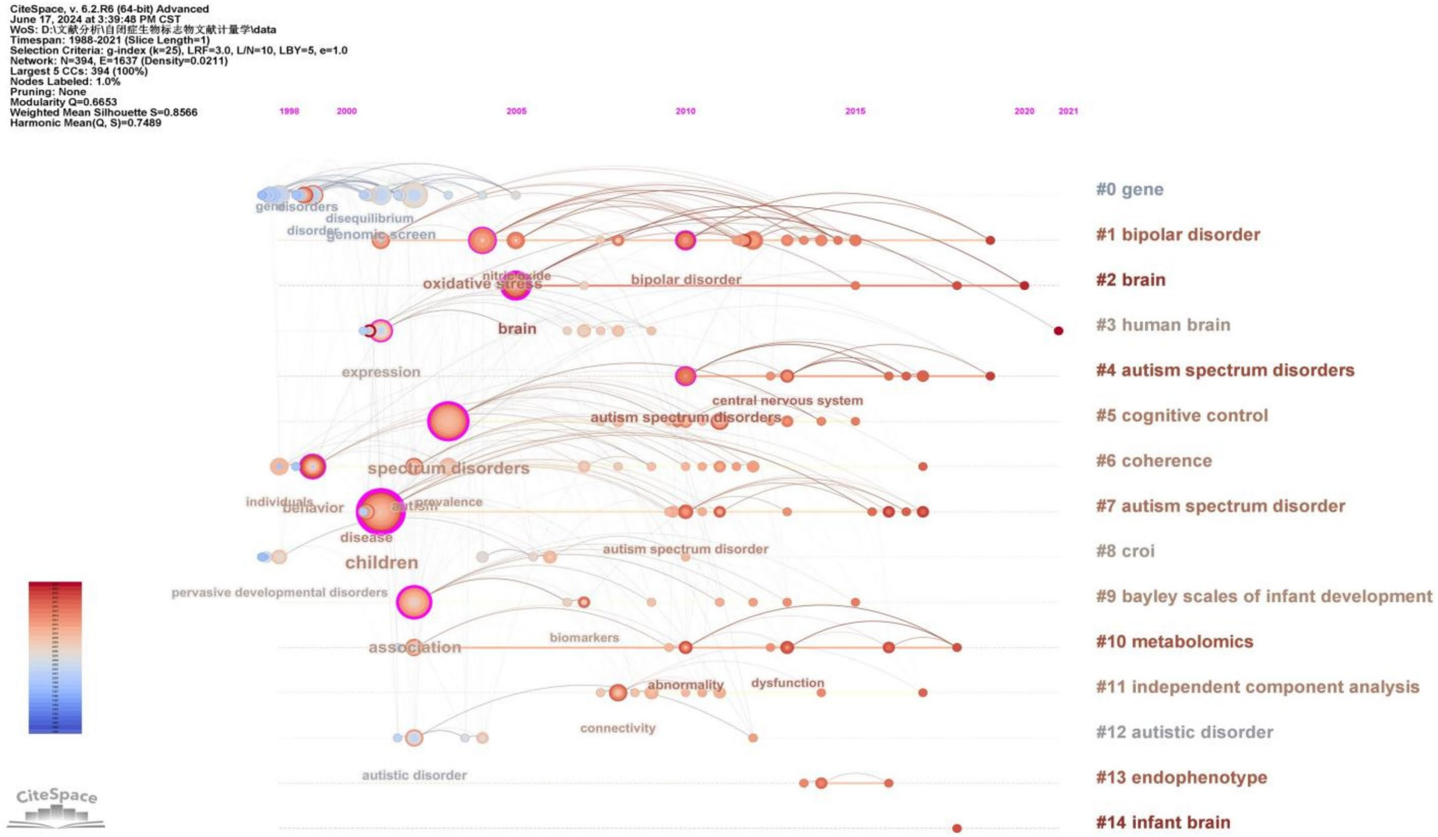
The timeline of the keyword cluster.

To gain deeper insight into the evolution of these clusters, we visualized the keyword cluster timeline. Figure 7 illustrates this timeline, analyzing the occurrence and duration of main keywords to uncover research hotspots in this field across different periods. Early studies predominantly focused on keywords such as “gene” (#0), “croi” (#8), and “coherence” (#6). Topics like “cognitive control” (#5), “Bayley Scales of Infant Development” (#9), “independent component analysis” (#11), and “endophenotype” (#13) were transient. In contrast, “bipolar disorder” (#1), “brain” (#2), “human brain” (#3), and “metabolomics” (#10) consistently remained prominent research topics and trends.

**Figure 8:**
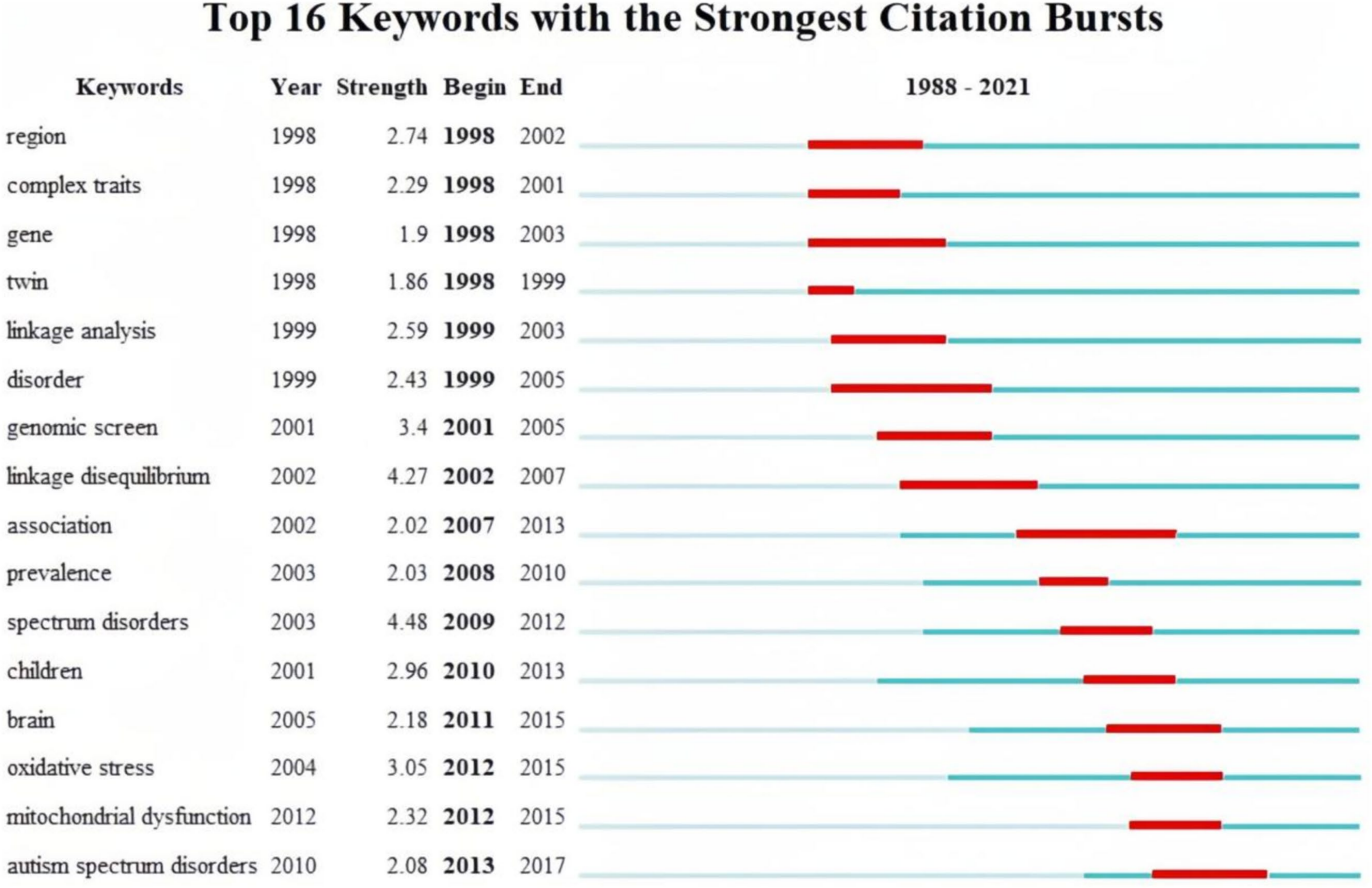
The top 16 keywords with the strongest citation bursts.

Finally, burst keywords were analyzed to identify keywords that garnered considerable attention over time. Figure 8 visualizes the top 16 keywords with the strongest bursts. The analysis revealed that keywords such as “region,” “gene,” “complex traits,” and “twin” exhibited early bursts, sustaining high-intensity interest from 1991 to 2007. Subsequently, keywords such as “linkage analysis,” “genomic screen,” and “linkage disequilibrium” gained prominence.

Since 2007, there has been a shift in focus toward keywords has shifted toward terms related to specific biochemical processes in the brains of children with autism. Notably, terms like “association,” “prevalence,” “children,” “brain,” “oxidative stress,” and “mitochondrial dysfunction” indicate a dedicated exploration of autism biomarkers through epidemiological characteristics.

## 4 Discussion

As the prevalence of autism spectrum disorder (ASD) continues to rise, the number of affected individuals has steadily increased, underscoring the growing impact of this condition on society (Maenner, 2021). Although there is no unified consensus on its exact mechanisms, factors such as genetics, brain function, and metabolites have all been implicated in autism’s development. Biomarkers, serving as indicators of biological processes or states, hold significant potential for diagnostic, therapeutic, and prognostic applications in the context of autism (Jensen et al., 2022).

This analysis of the 100 top-cited studies on autism biomarkers provides valuable insights for the research community. It offers a historical overview of the most influential studies, showcasing key trends and advancements. Additionally, it contributes to a deeper understanding of the landscape of autism biomarkers, which can aid researchers, clinicians, and policymakers in advancing interventions for autism spectrum disorders.

Beyond analyzing the basic characteristics of these articles, we conducted an in-depth examination of the highly cited studies to uncover the current state of autism biomarker research and identify emerging trends, providing our perspectives on the field’s development.

### 4.1 Basic characteristics of the 100 top-cited autism biomarkers researches

We analyzed the key characteristics of the 100 most-cited articles, including publication year, country, and leading authors, to provide insights for journals interested in identifying experienced authors and relevant research contributions in this field.

The growing number of articles on autism biomarkers reflects society’s increasing demand for greater attention and treatment of autism. International collaboration and the use of technology present promising opportunities to tackle this issue.

The growing trend of global collaboration in this field is both encouraging and promising. Collaborative initiatives bring together diverse expertise and resources, accelerating progress in ASD biomarker research. What we see is a growing cooperation between countries, with authors increasingly collaborating and forming networks of partnerships. Notably, the United States plays a dominant role in both research productivity and citation impact, highlighting its global leadership in advancing our collective understanding of ASD. Beyond the collaboration with researchers from the developed countries, US scholars are also working with experts from developing nations, such as China, to establish a global network focused on autism biomarkers (Liu et al., 2001; Morrow et al., 2008; Voineagu et al., 2011).

In examining the basic characteristics of highly cited autism biomarker studies, we found that technology plays a pivotal role in advancing autism biomarker research. Our analysis reveals that the majority of influential articles were published between 2010 and 2020. Further examination reveals that since 2010, the introduction and advancement of technologies in neuroimaging, genomics, and molecular biology—such as high-resolution brain imaging, diffusion tensor imaging (DTI), magnetoencephalography (MEG), gene sequencing, and bioinformatics—have provided powerful tools for the in-depth exploration of autism biomarkers. The United States benefits from advanced technology, especially with its cutting-edge scientific institutions such as Harvard University and Massachusetts General Hospital, placing it in a leading position that clearly demonstrates this point.

### 4.2 Current research hotspots

In bibliometrics, keywords serve as highly generalized representations of an article, with high-frequency keywords specifically employed to identify focal points and cutting-edge areas in a research field. In this study, we conducted an in-depth analysis of the top keywords, exploring their clusters and tracing the evolution of these clusters over time. Through this process, we unveiled the current progress in the field of autism biomarker research and identified potential future research trends.

Recent research on biomarkers for autism has mainly focused on four key areas: biomarkers in childhood, genetic markers of autism, oxidative stress markers, and mitochondrial dysfunction.

#### 4.2.1 Biomarkers in childhood

The keyword “children” appears most frequently, indicating that researchers aim to investigate biomarkers from early childhood. Among the 100 most-cited articles, nearly 40% of the studies focused on the exploring the biomarkers in childhood. Studying autism biomarkers in early childhood may be due to several reasons. Autism typically manifests and is diagnosed during childhood, which is also when symptoms are most pronounced. Identifying biomarkers during this period can aid in early diagnosis (Chawarska et al., 2007; Ozonoff et al., 2011; Lord et al., 2018). Additionally, the complex etiology of autism, involving the interaction of genetic and environmental factors, makes it easier to obtain accurate data by studying childhood biomarkers, minimizing interference from other factors. This helps us better understand the origins, development, and trends of autism (Hallmayer et al., 2011; Gaugler et al., 2014; Sandin et al., 2014). Moreover, certain biomarkers may be more stable in childhood, as the nervous and physiological systems are not fully developed, making changes in biomarkers easier to detect. Researching childhood biomarkers can also reveal key pathways and targets in the pathophysiology of autism, enabling early intervention and treatment, and thereby reducing future burdens for individuals with autism.

The detection of biomarkers in childhood has become an effective way to predict autism in adulthood. Researches show that genetic, neuroimaging, and biochemical markers from childhood provide significant predictive accuracy for adult outcomes. For example, genetic variations such as FMR1 mutations or 16q11.2 copy number variations increase the likelihood of severe neurodevelopmental challenges in adulthood (Stessman et al., 2014; Sandin et al., 2017). Neuroimaging studies reveal weakened functional connectivity in the default mode network of autistic children, persisting into adulthood and predicting social difficulties. In summary, exploring childhood biomarkers is crucial for predicting adult autism severity and enabling early interventions.

#### 4.2.2 Genetic biomarkers

Significant progress has been made in the genetic research of autism biomarkers, particularly in the fields of genes (keywords cluster #0), chromosomes, and cytogenetics (keywords cluster #8). Many studies on autism’s genetic causes have employed genome-wide screening to identify shared genetic markers within families. By analyzing genetic patterns in parents and siblings, researchers aim to uncover hereditary factors contributing to autism. Studies confirm a correlation between the incidence of autism and genetics, with family genetic loci and genes acting as hereditary factors in children with autism (Philippe et al., 1999; Risch et al., 1999; Muhle et al., 2004; Morrow et al., 2008; Anney et al., 2010). Many genes are associated with autism spectrum disorders, and copy number variations and rare gene mutations (e.g., SHANK3, NRXN1, CHD8, SCN2A) are closely linked to autism (Glessner et al., 2009). Regarding chromosomes, research shows that abnormalities such as duplications or deletions in the chromosomes of individuals with autism significantly increase the prevalence of the disorder, especially the duplication of chromosome 15q11-13 (Cook et al., 1998; Philippe et al., 1999; Buxbaum et al., 2002; Shen et al., 2010). Furthermore, variations in susceptibility regions like 7q22-q31, 2q24.3-q31, and 11p12-p13 affect neurodevelopment through multiple mechanisms, increasing the risk of autism (Consortium I.M.G.S.o.A, 1998, 2001; Muhle et al., 2004; Vorstman et al., 2006).

Related studies also reveal a correlation between the occurrence of autism and the interaction of genetic and environmental factors, as well as interactions between genes. For instance, the review “The genetics of autism” indicates that interactions among multiple genes lead to idiopathic autism, while epigenetic factors and exposure to environmental modulators may contribute to the variable expression of autism-related traits (Muhle et al., 2004; Rossignol and Frye, 2012b; Rossignol et al., 2014).

#### 4.2.3 Oxidative stress markers

Among the 100 top-cited articles, 14 articles focus on clarifying the relationship between ASD and oxidative stress. Oxidative stress is a physiological state caused by an imbalance between the production and clearance of Reactive Oxygen Species (ROS) and Reactive Nitrogen Species (RNS) in the body (Chauhan and Chauhan, 2006; Frustaci et al., 2012). This imbalance is thought to be closely related to the pathological mechanisms of various neurodevelopmental disorders.

Glutathione is a crucial antioxidant in the human body, playing a significant role in maintaining cellular redox balance. Abnormal glutathione metabolism is considered an important component of the pathological mechanism of autism. Studies have found that the total amount of glutathione, including reduced glutathione (GSH) and oxidized glutathione (GSSG) in patients with autism is often reduced. Specifically, the level of GSH is significantly reduced, while the level of GSSG is relatively elevated. This results in a decreased GSH/GSSG ratio, reflecting an imbalance in the cellular redox state. Changes in this ratio are considered an important marker of oxidative stress (James et al., 2004; Frustaci et al., 2012; Rose et al., 2012). Research on glutathione-related enzymes, such as glutathione synthase (GSS), glutathione reductase (GR), and glutathione peroxidase (GPx), reveals their key roles in glutathione synthesis, regeneration, and functional maintenance (Frustaci et al., 2012; Rose et al., 2012; Gu et al., 2015). In autism patients, enzyme activities, such as reduced GPx and GR activities, are often abnormal, resulting in impaired GSH regeneration and exacerbated oxidative stress.

In terms of identifying and validating oxidative stress markers, researchers have identified markers that show significant changes in individuals with autism (Chauhan et al., 2012). These specific biomolecules, including Malondialdehyde (MDA), 4-hydroxynonenal (4-HNE), and protein carbonyl, indicate the degree of lipid peroxidation and protein oxidation, respectively. Changes in the concentrations of these markers in blood, urine, and cerebrospinal fluid are thought to reflect the oxidative stress status of individuals with autism, providing important indicators for clinical diagnosis and disease assessment (Frustaci et al., 2012). Advanced technologies such as High-Performance Liquid Chromatography (HPLC), Gas Chromatography–Mass Spectrometry (GC–MS), and Enzyme-Linked Immunosorbent Assay (ELISA) have made the quantitative detection of these markers more accurate and sensitive, providing reliable data for studying oxidative stress in autism (Yao et al., 2006; Chauhan et al., 2012; El-Ansary and Al-Ayadhi, 2012).

Evaluating antioxidant enzyme activity is another critical aspect. Antioxidant enzymes, including Superoxide Dismutase (SOD), Catalase (CAT), and Glutathione Peroxidase (GPx), play a crucial role in scavenging reactive oxygen species and maintaining redox balance in the body (Chauhan et al., 2012; Frustaci et al., 2012). Studies have found that the activity of these enzymes often changes in individuals with autism, manifesting as either decreased or abnormally increased activity. Assessing changes in antioxidant enzyme activity provide further insights into the oxidative stress status of individuals with autism.

#### 4.2.4 Mitochondrial dysfunction

Mitochondrial dysfunction leads to energy metabolism disorders and excessive production of reactive oxygen species, triggering oxidative stress (Rossignol and Frye, 2012a; Rossignol and Frye, 2014). In autistic patients, research on mitochondrial dysfunction focuses on mitochondrial DNA, bioenergetics, and mitochondrial autophagy.

Mitochondria have a genome independent of nuclear DNA and are more susceptible to mutations. Many studies have found multiple mutations and variations in mitochondrial DNA in individuals with autism, affecting mitochondrial function and leading to energy metabolism disorders. Studies have validated that point mutations, deletions, and copy number variations in mtDNA are all associated with the severity of autism symptoms (Giulivi et al., 2010; Rossignol and Frye, 2012a).

Additionally, mitochondria in autistic individuals often exhibit abnormalities in ATP synthesis, redox response, and electron transport chain (ETC) function. Studies on mitochondrial function in cells and tissues of autistic individuals have found significant differences in oxidative phosphorylation efficiency, membrane potential, and ROS production. These dysfunctions may lead to insufficient energy supply to nerve cells, affecting neurodevelopment and function, thereby increasing the risk of autism (Giulivi et al., 2010; Rossignol and Frye, 2012a).

The relationship between mitochondria and autophagy has also garnered research attention. Autophagy is a crucial process for cells to clear damaged mitochondria and maintain intracellular stability. Numerous studies have found that autophagy function in autistic individuals may be impaired, resulting in the accumulation of damaged mitochondria, which produce more ROS and exacerbate oxidative stress (Tang et al., 2014).

### 4.3 Potential research trends

Based on the evolution of keyword clustering, we believe that future research directions for autism biomarkers may focus on brain studies, the comorbidity of autism with other psychiatric disorders, and metabolic markers.

#### 4.3.1 Further brain research

The keyword “brain” has become the second most prominent keyword and the second-largest cluster. The timeline of keyword clustering shows that this is currently the hottest topic, indicating that brain-related research will undoubtedly continue to be a major focus in the future. Current research on the brain mechanisms of autism primarily employs techniques such as functional magnetic resonance imaging (fMRI), structural magnetic resonance imaging (sMRI), electroencephalography (EEG), magnetoencephalography (MEG) and event-related potentials (ERP) to uncover neuroimaging biomarkers for autism (Calhoun et al., 2008; Schumann et al., 2010; Abraham et al., 2017; Gilmore et al., 2018). These methods will continue to evolve, enhancing our understanding of both the structure and function of the autistic brain.

Brain research may be developed from several aspects, including deepening our understanding of brain structure, with an emphasis on connectivity and functional integration within brain networks. Most importantly, it will aim to establish a multimodal model to comprehensively examine the brain mechanisms in individuals with autism.

Current neuroimaging research has provided a substantial amount of preliminary data on the brain structure and function of autism patients, which still needs to be expanded. Using structural magnetic resonance imaging (sMRI), voxel-based morphometric analysis has found that individuals with autism exhibit increased white matter volume in the temporal and frontal lobes, as well as enlarged gray matter volumes in regions such as the postcentral gyrus and superior temporal gyrus. Cortical folding analysis suggests an initial increase in cortical folds in some brain areas, which decreases with age (Jiao et al., 2010; Ecker et al., 2015). Functional MRI (fMRI) reveals that autistic individuals show heightened activity in brain regions related to visual perception and pattern recognition, but lower activity in areas related to social and emotional processing (Di Martino et al., 2009; Schipul et al., 2011). Additionally, resting-state fMRI analysis of striatal connectivity reveals changes, such as decreased connectivity between the striatum and prefrontal cortex, potentially disrupting normal neural communication (Yerys et al., 2015). While there have been significant findings regarding the brain regions involved in autism, the brain remains a complex and mysterious organ. Investigating the potential underlying brain mechanisms in individuals with autism is an ongoing challenge and an exciting area of research. Understanding these mechanisms could provide deeper insights into the condition and open new avenues for more effective treatments and interventions. This remains a key interest for researchers in the field.

However, a major unresolved issue is that current research primarily relies on single techniques, each providing only a partial view of the brain. The challenge lies in integrating multimodal data and combining different technologies to more comprehensively and accurately reveal the neurobiological mechanisms of autism. Differences in the spatial and temporal resolution, as well as measurement indicators, between modalities highlight the need for new data analysis methods and models to achieve this integration. Developing such approaches will be a crucial breakthrough in autism brain research.

#### 4.3.2 Comorbidity of autism and other psychiatric disorders

Autism does not exist in isolation; many autism patients also suffer from other psychiatric disorders such as attention deficit hyperactivity disorder (ADHD), anxiety disorders, depression, and epilepsy (Gillberg and Billstedt, 2000; Simonoff et al., 2008; Joshi et al., 2013; Lai et al., 2019). This comorbidity phenomenon not only increases the complexity of diagnosis and treatment but also poses greater challenges for patients and their families. Therefore, studying the comorbidity of autism with other psychiatric disorders is important for understanding the pathological mechanisms of autism and developing effective treatment strategies.

The most cited article “Identification of risk loci with shared effects on five major psychiatric disorders: A genome-wide analysis,” explores specific genetic variants’ effects on autism, attention deficit disorder, bipolar disorder, major depression, and schizophrenia. It identifies chromosome 3p21 and10q24, along with single nucleotide polymorphisms (SNPs) within two L-type voltage-gated calcium channel subunits, CACNA1C and CACNB2, as potential biomarkers for these disorders (Consortium C.-D.G.o.t.P.G, 2013). This underscores the research focus on common biomarkers across different psychiatric conditions. Therefore investigating shared biomarkers of autism in conjunction with other psychiatric disorders represents a significant research trend and hotspot moving forward.

The presence of multiple comorbid conditions makes accurate diagnosis challenging. For instance, when autism co-occurs with Attention Deficit Hyperactivity Disorder (ADHD), the typical ADHD symptoms of inattention, hyperactivity, and impulsivity can overlap and become confounded with autism’s own traits, such as distractibility and stereotyped behaviors. This overlap makes it difficult for clinicians to distinguish between the two based solely on external behavioral manifestations, often necessitating more comprehensive and in-depth assessment methods for accurate diagnosis. The high prevalence of comorbidity between autism and other psychiatric disorders suggests that these disorders may share common pathological bases. For example, both autism and ADHD involve brain function abnormalities, particularly in executive function, attention, and behavioral control (Castellanos and Proal, 2012). By studying these comorbidity phenomena, current research has begun to explore common neurobiological mechanisms between autism and other psychiatric disorders (Crespi et al., 2010; Consortium C.-D.G.o.t.P.G, 2013; Smaga et al., 2015).

Additionally, the challenges posed by comorbidities are even more pronounced in treatment. The comorbidity research can improve the diagnosis and treatment of autism. For patients with both autism and other psychiatric disorders, single-treatment methods are often ineffective. Understanding the comorbidity mechanisms of these disorders allows doctors to develop multi-level and multi-target treatment strategies, enhancing treatment efficacy (Matson and Goldin, 2013; Vasa and Mazurek, 2015).

Future research on autism biomarkers will focus on the comorbidity of autism with other psychiatric disorders, revealing common pathological mechanisms and promoting the development of new diagnostic and therapeutic methods, improving early detection and intervention capabilities for these complex disorders. Through comorbidity research, we hope to better understand and address autism and its associated psychiatric disorders (Loth et al., 2016; Frye et al., 2019).

#### 4.3.3 Metabolic markers

In recent years, an increasing number of studies have found metabolic abnormalities in autism patients (Adams et al., 2011a; Adams et al., 2011b; Kang et al., 2018). These abnormalities involve various metabolic pathways, including energy metabolism, amino acid metabolism, lipid metabolism, and oxidative stress (Rossignol and Frye, 2014; Frye et al., 2019).

Although genetic factors play a significant role in the development of autism, the impact of environmental factors cannot be overlooked. Metabolic processes act as a crucial interface between genes and the environment. Investigating metabolic biomarkers in children with autism provides deeper insights into the condition’s pathogenesis. For example, metabolic biomarkers can reveal abnormalities in processes such as cellular energy metabolism and neurotransmitter synthesis. These disruptions may serve as potential underlying causes of impaired brain development in children with autism.

As mentioned above, some studies have found mitochondrial dysfunction in autism patients, leading to energy metabolism disorders (Rossignol and Frye, 2012b; Morris and Berk, 2015); other studies have shown elevated oxidative stress levels in autism patients, which may be related to neuronal damage (James et al., 2004; Ng et al., 2008; Frustaci et al., 2012). By studying these metabolic markers, scientists can better understand the biological basis of autism. Metabolic marker research provides an objective biological testing method, improving diagnostic accuracy and early detection capabilities. For instance, scientists can analyze metabolite levels in the blood, urine, or other body fluids of autism patients to find specific metabolic markers, assisting in the diagnosis of autism and providing targeted treatment (Gabriele et al., 2014; Rossignol and Frye, 2014).

### 4.4 Critical insights and reflections of potential trend

We conducted an in-depth analysis of each article, carefully evaluating the current state and trends in research as discussed above. Based on this, we offer our critical insights and reflections on biomarker studies in autism accordingly. We believe these analyses will help advance the field further and offer new perspectives for the diagnosis and treatment of autism.

#### 4.4.1 Application of emerging technologies for autism biomarkers

In analyzing the top 100 highly-cited studies, we found that autism biomarker research, especially concerning genetic and neuroimaging biomarkers in autism patients, heavily relies on the advancement and application of cutting-edge technologies. Current autism biomarker detection predominantly involves established techniques like fMRI, sMRI, EEG, and MEG (Calhoun et al., 2008; Schumann et al., 2010; Abraham et al., 2017; Gilmore et al., 2018). With innovations in artificial intelligence and machine learning technologies, we believe the application of new technologies is a promising direction, particularly in the research of the diagnosis of autism biomarkers.

Artificial intelligence (AI) and machine learning demonstrate considerable application value in the diagnosis of autism biomarkers. Based on the precision and automation of AI and machine learning, these technologies can be applied to large-scale whole genome sequencing data to uncover complex genetic variation patterns associated with autism (Nisar and Haris, 2023; Maqsood et al., 2024). They can also be used to process vast amounts of MRI and fMRI data to capture and analyze complex relationships in brain structural data. These approaches are far more challenging with traditional manual methods, but AI and machine learning not only save time and effort but also provide higher accuracy (do Rêgo and Araújo-Filho, 2024; Maqsood et al., 2024; Wankhede et al., 2024).

Regarding genetic biomarkers diagnosis, autism-related genes such as SHANK3, NRXN1, and CNTNAP2 exhibit significant complexity and variability, with mutations appearing as single nucleotide polymorphisms (SNPs), small insertions or deletions (Indels), and copy number variations (CNVs). Deep learning algorithms like Convolutional Neural Networks (CNNs) and Recurrent Neural Networks (RNNs) can automatically extract more refined features from large datasets, making them more efficient and accurate than manual analysis (Nisar and Haris, 2023; Maqsood et al., 2024). From a brain structural perspective, AI and machine learning can analyze vast datasets through clustering methods to identify abnormalities in specific brain regions and autism-specific structural features (Khodatars et al., 2021; Bahathiq et al., 2022). For example, clustering analyses of gray matter volume data in autism patients and control groups can reveal the relationship between reduced frontal lobe gray matter volume and executive function deficits, as well as abnormal temporal lobe gray matter volume and social cognitive impairments.

Additionally, AI and machine learning provide the opportunity for dynamic tracking of biomarkers at different time points and stages, allowing for the creation of evolving pathways for biomarkers. By integrating data from other biological markers such as metabolic and genetic markers, the accuracy of disease diagnosis and prediction can be improved, providing a basis for timely adjustments to treatment plans and enhancing treatment outcomes for autism patients.Thus, we believe that the use of emerging technologies will undoubtedly become a focal point in future research.

#### 4.4.2 Longitudinal study of autism biomarkers

We analyzed key areas of biomarker research in childhood autism, emphasizing its importance for early diagnosis, detection, and predictive studies. However, the continuous changes in autism biomarkers throughout development remain unclear. Current research often focuses on childhood and adulthood biomarkers separately, leaving gaps in understanding their evolution and longitudinal dynamics. Autism, as a lifelong neurodevelopmental disorder, involves symptoms and pathological characteristics that vary with age and developmental stage (Seltzer et al., 2004; Zwaigenbaum et al., 2013).

Longitudinal studies, which repeatedly measure the same group of individuals at multiple time points, can offer a more comprehensive and in-depth understanding. Through long-term tracking, researchers can observe changes in biomarkers during disease progression, understand their role in the disease course, and identify early predictors of autism (Singer, 2003; Ecker et al., 2015). However, there is currently a shortage of longitudinal studies, especially those that span the lifespan, investigating autism biomarkers.

The dynamic progression of autism biomarkers is indeed essential to understanding the disorder comprehensively. For instance, studies have identified a strong correlation between mutations in the FMR1 gene and autism (Hagerman et al., 2018; Richter and Zhao, 2021). However, longitudinal research on how such genetic anomalies influence autism phenotypes across developmental stages remains limited. While we know that mutations in FMR1 lead to the loss or dysfunction of its encoded protein, the precise mechanisms by which this anomaly affects brain development and behavioral symptoms from infancy to adulthood remain unclear (Verkerk et al., 1991).

Similarly, the dynamic interplay between multiple autism-related genes (e.g., SHANK3, PTEN) and their joint regulation of brain development and behavior over time has yet to be thoroughly investigated (Monteiro and Feng, 2017). Additionally, while glutamate level abnormalities have been observed in individuals with autism, studies have not examined how these levels change with age. For example, elevated glutamate levels detected in early childhood are not yet understood—whether they represent a transient developmental fluctuation or a persistent neurodevelopmental factor influencing adult social and cognitive symptoms remains unknown (Robertson and Baron-Cohen, 2017; Horder et al., 2018).

The brain’s plasticity underscores the significance of dynamic changes and developmental processes. While exploring brain mechanisms at a single time point, such as during childhood, is valuable, understanding the trajectory of biomarker evolution under this plasticity is crucial. Future research should prioritize studying the dynamic progression of biomarkers alongside current cross-sectional approaches. Although such longitudinal studies are time-and resource-intensive, they hold immense potential for deepening our understanding of autism mechanisms and advancing effective treatments.

#### 4.4.3 Specificity of autism biomarkers

Current research trends indicate a trend toward studying the comorbidity of autism with other psychiatric disorders (Gillberg and Billstedt, 2000; Simonoff et al., 2008; Joshi et al., 2013; Lai et al., 2019). However, due to the unique nature of autism, we believe that specific research on autism biomarkers is important. The specific study of autism biomarkers is crucial for more accurate diagnosis of the disorder itself.

To understand the core pathological mechanisms of autism, while the study of comorbid biomarkers can shed light on their association with other disorders, it cannot replace the need to explore autism’s intrinsic pathology. Comorbid biomarkers may blur diagnostic boundaries, whereas autism-specific biomarkers—such as mutations in SHANK3, NRXN1, and CHD8—are directly tied to autism, but may also be found in other conditions like intellectual disabilities and ADHD (Zoghbi and Bear, 2012; De Rubeis and Buxbaum, 2015). Therefore, they cannot be solely used for initial diagnosis. From a personalized treatment standpoint, autism-specific biomarkers provide targeted therapeutic strategies, enhancing the potential for improving core symptoms and quality of life.

Therefore, future research should not only explore the comorbidity of autism with other psychiatric disorders but also focus on advancing the specific study of autism biomarkers. This will enable more precise diagnosis of autism’s core features, uncover its unique pathogenesis, and guide personalized treatment strategies. Future research should aim to develop more specific and sensitive biomarkers, which may require the integration of multiple biological data, such as genomic, metabolomic, and imaging data. The combined application of multiple biomarkers could enhance diagnostic specificity and sensitivity, despite increasing research and application complexity. Additionally, large-scale validation studies are needed to ensure the reliability of these biomarkers in different populations and clinical settings. The application of new technologies such as machine learning and artificial intelligence may help identify more precise diagnostic patterns from complex data.

## 5 Limitations

While selecting the 100 most cited references provides a foundation of widely recognized work, we acknowledge that this approach may have limitations.

Firstly, we exclusively relied on the Web of Science database for our analysis and did not encompass data from other databases such as Scopus, Medline, and Google Scholar. Consequently, some essential papers indexed by alternative databases might have been overlooked. Secondly, the dynamic nature of citation counts over time implies that the composition of the 100 top-cited articles is subject to change. Thirdly, citation rates are affected by a multitude of factors, many of which extend beyond the scope of this study. While citation analysis serves as a valuable metric for recognition, it may not be the optimal measure for assessing the quality or significance of scientific research. Finally, the tendency of citation analysis to undervalue newly published studies due to the inherent advantage of older studies in accumulating citations is acknowledged. Therefore, it may inadvertently reinforce established ideas and overlook innovative studies that have yet to gain citation momentum. Such circularity could limit the exploration of emerging perspectives and novel hypotheses.

In future studies, we plan to expand the database inclusion and implement dynamic tracking to monitor changes over time. We also recommend that researchers conduct a thorough review of all relevant literature to analyze valuable yet under-cited articles. Despite these constraints, as the inaugural citation analysis in autism, we posit that our findings will augment the comprehension of trends and classic publications in this field.

## 6 Conclusion

In the present bibliometric study, we identified and analyzed the 100 top-cited publications on autism biomarkers, examining key aspects such as publication years, document types and categories, journals, countries, institutes, authors, and keywords. We also conducted an analysis of current research hotspots and future research trends. The current research hotspots on autism biomarkers mainly focus on genetic markers of autism, childhood biomarkers, oxidative stress markers, and mitochondrial dysfunction, and future research may continue to deepen from “brain,” comorbidity of autism with other psychiatric disorders, and metabolic markers. Based on the current research reflection, we believe that studies on application of emerging technologies of autism biomarkers, longitudinal study of autism biomarkers and specificity of autism biomarkers will make greater contributions to autism biomarker research. These comprehensive insights into the most impactful studies in the field of autism biomarkers aim to assist doctors, researchers, and other stakeholders in enhancing their understanding of prevailing trends and influential contributions to autism research.

## Footnotes

^1^ www.webofscience.com

^2^ https://JCR.Clarivate.com/
