# Supplementary material for: Mapping the structure of biomarkers in autism spectrum disorder: a review of the most influential studies
Source: Front Neurosci. 2024 Dec 13;18:1514678. doi: 10.3389/fnins.2024.1514678 (PMC11671500; doi:10.3389/fnins.2024.1514678)
Supplement: Supplementary file 1 [file Table_1.docx]

**Table 1** The 100 top-cited articles in autism biomarkers, ordered by number of citations (2080-180)

| Rank | Title | First Author | Journal | Publication Year | Total citations | Citation per year |
| --- | --- | --- | --- | --- | --- | --- |
| 1 | Identification of risk loci with shared effects on five major psychiatric disorders: A genome-wide analysis | Smoller, J. W. | LANCET | 2013 | 2088 | 189.82 |
| 2 | Consensus statement: Chromosomal microarray is a first-tier clinical diagnostic test for individuals with developmental disabilities or congenital anomalies | Miller, D. T. | AMERICAN JOURNAL OF HUMAN GENETICS | 2010 | 1883 | 134.50 |
| 3 | Transcriptomic analysis of autistic brain reveals convergent molecular pathology | Voineagu, I. | NATURE | 2011 | 1316 | 101.23 |
| 4 | Autism genome-wide copy number variation reveals ubiquitin and neuronal genes | Glessner, J. T. | NATURE | 2009 | 1020 | 68.00 |
| 5 | The genetics of autism | Muhle, R. | PEDIATRICS | 2004 | 812 | 40.60 |
| 6 | Hypoplasia of cerebellar vermal lobule VI and VII in autism | Courchesne, E. | NEW ENGLAND JOURNAL OF MEDICINE | 1988 | 795 | 22.08 |
| 7 | Using Support Vector Machine to identify imaging biomarkers of neurological and psychiatric disease: A critical review | Orrù, G. | NEUROSCIENCE AND BIOBEHAVIORAL REVIEWS | 2012 | 717 | 59.75 |
| 8 | Metabolic biomarkers of increased oxidative stress and impaired methylation capacity in children with autism | James, S. J. | AMERICAN JOURNAL OF CLINICAL NUTRITION | 2004 | 663 | 33.15 |
| 9 | Gastrointestinal flora and gastrointestinal status in children with autism-comparisons to typical children and correlation with autism severity | Adams, J. B. | BMC GASTROENTEROLOGY | 2011 | 652 | 50.15 |
| 10 | Oxidative stress in psychiatric disorders: evidence base and therapeutic implications | Ng, F | INTERNATIONAL JOURNAL OF NEUROPSYCHOPHARMACOLOGY | 2008 | 625 | 39.06 |
| 11 | Sensory processing in autism: A review of neurophysiologic findings | Marco, E. J. | PEDIATRIC RESEARCH | 2011 | 601 | 46.23 |
| 12 | Identifying autism loci and genes by tracing recent shared ancestry | Morrow, E. M. | SCIENCE | 2008 | 550 | 34.38 |
| 13 | Mitochondrial dysfunction in autism spectrum disorders: a systematic review and meta-analysis | Rossignol, D. A. | MOLECULAR PSYCHIATRY | 2012 | 542 | 45.17 |
| 14 | A genomic screen of autism: Evidence for a multilocus etiology | Risch, N. | AMERICAN JOURNAL OF HUMAN GENETICS | 1999 | 533 | 21.32 |
| 15 | Autism | Levy, S. E. | LANCET | 2009 | 510 | 34.00 |
| 16 | Organophosphate pesticide exposure and neurodevelopment in young Mexican-American children | Eskenazi, B. | ENVIRONMENTAL HEALTH PERSPECTIVES | 2007 | 502 | 29.53 |
| 17 | A functional genetic link between distinct developmental language disorders | Vernes, S. C. | NEW ENGLAND JOURNAL OF MEDICINE | 2008 | 488 | 30.50 |
| 18 | Patches of disorganization in the neocortex of children with autism | Stoner, R. | NEW ENGLAND JOURNAL OF MEDICINE | 2014 | 487 | 48.70 |
| 19 | An overview of autism spectrum disorder, heterogeneity and treatment options | Masi, A. | NEUROSCIENCE BULLETIN | 2017 | 478 | 68.29 |
| 20 | A genome-wide linkage and association scan reveals novel loci for autism | Weiss, L. A. | NATURE | 2009 | 477 | 31.80 |
| 21 | Modulation of temporally coherent brain networks estimated using ICA at rest and during cognitive tasks | Calhoun, V. D. | HUMAN BRAIN MAPPING | 2008 | 460 | 28.75 |
| 22 | Imaging structural and functional brain development in early childhood | Gilmore, J. H. | NATURE REVIEWS NEUROSCIENCE | 2018 | 450 | 75.00 |
| 23 | Oxytocin and social affiliation in humans | Feldman, R. | HORMONES AND BEHAVIOR | 2012 | 450 | 37.50 |
| 24 | A genome-wide scan for common alleles affecting risk for autism | Anney, R. | HUMAN MOLECULAR GENETICS | 2010 | 446 | 31.86 |
| 25 | A full genome screen for autism with evidence for linkage to a region on chromosome 7q | Bailey, A. | HUMAN MOLECULAR GENETICS | 1998 | 433 | 16.65 |
| 26 | Review of advanced techniques for the estimation of brain connectivity measured with EEG/MEG | Sakkalis, V. | COMPUTERS IN BIOLOGY AND MEDICINE | 2011 | 429 | 33.00 |
| 27 | Positive association of the oxytocin receptor gene (OXTR) with autism in the Chinese Han population | Wu, S. P. | BIOLOGICAL PSYCHIATRY | 2005 | 419 | 22.05 |
| 28 | Salience network-based classification and prediction of symptom severity in children with autism | Uddin, L. Q. | JAMA PSYCHIATRY | 2013 | 416 | 37.82 |
| 29 | Longitudinal magnetic resonance imaging study of cortical development through early childhood in autism | Schumann, C. M. | JOURNAL OF NEUROSCIENCE | 2010 | 397 | 28.36 |
| 30 | Genome-wide scan for autism susceptibility genes | Philippe, A. | HUMAN MOLECULAR GENETICS | 1999 | 391 | 15.64 |
| 31 | Developmental pathways to autism: A review of prospective studies of infants at risk | Jones, E. J. | NEUROSCIENCE AND BIOBEHAVIORAL REVIEWS | 2014 | 377 | 37.70 |
| 32 | A review of research trends in physiological abnormalities in autism spectrum disorders: immune dysregulation, inflammation, oxidative stress, mitochondrial dysfunction and environmental toxicant exposures | Rossignol, D. A. | MOLECULAR PSYCHIATRY | 2012 | 372 | 31.00 |
| 33 | A genomewide screen for autism: Strong evidence for linkage to chromosomes 2q, 7q, and 16p | Palferman, S. | AMERICAN JOURNAL OF HUMAN GENETICS | 2001 | 371 | 16.13 |
| 34 | Adult brain and behavioral pathological markers of prenatal immune challenge during early/middle and late fetal development in mice | Meyer, U. | BRAIN BEHAVIOR AND IMMUNITY | 2008 | 370 | 23.13 |
| 35 | Deriving reproducible biomarkers from multi-site resting-state data: An Autism-based example | Abraham, A. | NEUROIMAGE | 2017 | 369 | 52.71 |
| 36 | Microglial activation in young adults with autism spectrum disorder | Suzuki, K. | JAMA PSYCHIATRY | 2013 | 337 | 30.64 |
| 37 | Association of the oxytocin receptor gene (OXTR) in Caucasian children and adolescents with autism | Jacob, S. | NEUROSCIENCE LETTERS | 2007 | 327 | 19.24 |
| 38 | Evidence of oxidative damage and inflammation associated with low glutathione redox status in the autism brain | Rose, S | TRANSLATIONAL PSYCHIATRY | 2012 | 320 | 26.67 |
| 39 | The 2nd to 4th digit ratio and autism | Manning, J. T. | DEVELOPMENTAL MEDICINE AND CHILD NEUROLOGY | 2001 | 319 | 13.87 |
| 40 | The serotonin system in autism spectrum disorder: From biomarker to animal models | Muller, C. L. | NEUROSCIENCE | 2016 | 316 | 39.50 |
| 41 | Cytokine aberrations in autism spectrum disorder: A systematic review and meta-analysis | Masi, A. | MOLECULAR PSYCHIATRY | 2015 | 313 | 34.78 |
| 42 | Epidemiologic studies of exposure to prenatal infection and risk of schizophrenia and autism | Brown, A. S. | DEVELOPMENTAL NEUROBIOLOGY | 2012 | 311 | 25.92 |
| 43 | EEG complexity as a biomarker for autism spectrum disorder risk | Bosl, W. | BMC MEDICINE | 2011 | 308 | 23.69 |
| 44 | Resting state EEG abnormalities in autism spectrum disorders | Wang, J. | JOURNAL OF NEURODEVELOPMENTAL DISORDERS | 2013 | 301 | 27.36 |
| 45 | Environmental toxicants and autism spectrum disorders: A systematic review | Rossignol, D. A. | TRANSLATIONAL PSYCHIATRY | 2014 | 299 | 29.90 |
| 46 | An autosomal genomic screen for autism | Barrett, S. | AMERICAN JOURNAL OF MEDICAL GENETICS | 1999 | 297 | 11.88 |
| 47 | Deficient autophagy in microglia impairs synaptic pruning and causes social behavioral defects | Kim, H. J. | MOLECULAR PSYCHIATRY | 2017 | 293 | 41.86 |
| 48 | Linkage-disequilibrium mapping of autistic disorder, with 15q11-13 markers | Cook, E. H. | AMERICAN JOURNAL OF HUMAN GENETICS | 1998 | 290 | 11.15 |
| 49 | Autism and maternally derived aberrations of chromosome 15q | Schroer, R. J. | AMERICAN JOURNAL OF MEDICAL GENETICS | 1998 | 288 | 11.08 |
| 50 | Sensitive parenting is associated with plasma oxytocin and polymorphisms in the OXTR and CD38 genes | Feldman, R. | BIOLOGICAL PSYCHIATRY | 2012 | 285 | 23.75 |
| 51 | Neuroimaging in autism spectrum disorder: brain structure and function across the lifespan | Ecker, C. | LANCET NEUROLOGY | 2015 | 274 | 30.44 |
| 52 | Identification of novel autism candidate regions through analysis of reported cytogenetic abnormalities associated with autism | Vorstman, J. A. | MOLECULAR PSYCHIATRY | 2006 | 274 | 15.22 |
| 53 | Transcriptome-scale spatial gene expression in the human dorsolateral prefrontal cortex | Maynard, K. R. | NATURE NEUROSCIENCE | 2021 | 266 | 88.67 |
| 54 | MMP-9 in translation: from molecule to brain physiology, pathology, and therapy | Vafadari, B. | JOURNAL OF NEUROCHEMISTRY | 2016 | 264 | 33.00 |
| 55 | Evidence linking oxidative stress, mitochondrial dysfunction, and inflammation in the brain of individuals with autism | Rossignol, D. A. | FRONTIERS IN PHYSIOLOGY | 2014 | 263 | 26.30 |
| 56 | Identification of significant association and gene-gene interaction of GABA receptor subunit genes in autism | Ma, D. Q. | AMERICAN JOURNAL OF HUMAN GENETICS | 2005 | 263 | 13.84 |
| 57 | Nutritional and metabolic status of children with autism vs. neurotypical children, and the association with autism severity | Adams, J. B. | NUTRITION & METABOLISM | 2011 | 261 | 20.08 |
| 58 | Clinical genetic testing for patients with autism spectrum disorders | Shen, Y. P. | PEDIATRICS | 2010 | 258 | 18.43 |
| 59 | Cholinergic activity in autism: Abnormalities in the cerebral cortex and basal forebrain | Perry, E. K. | AMERICAN JOURNAL OF PSYCHIATRY | 2001 | 254 | 11.04 |
| 60 | Oxidative stress-related biomarkers in autism: Systematic review and meta-analyses | Frustaci, A. | FREE RADICAL BIOLOGY AND MEDICINE | 2012 | 252 | 21.00 |
| 61 | A genomewide screen for autism susceptibility loci | Liu, J. J. | AMERICAN JOURNAL OF HUMAN GENETICS | 2001 | 252 | 10.96 |
| 62 | DNA methylation of BDNF as a biomarker of early-life adversity | Kundakovic, M. | PROCEEDINGS OF THE NATIONAL ACADEMY OF SCIENCES OF THE UNITED STATES OF AMERICA | 2015 | 246 | 27.33 |
| 63 | Association between a GABRB3 polymorphism and autism | Buxbaum, J. D. | MOLECULAR PSYCHIATRY | 2002 | 244 | 11.09 |
| 64 | Linkage and association of the glutamate receptor 6 gene with autism | Jamain, S. | MOLECULAR PSYCHIATRY | 2002 | 243 | 11.05 |
| 65 | Validating γ oscillations and delayed auditory responses as translational biomarkers of autism | Gandal, M. J. | BIOLOGICAL PSYCHIATRY | 2010 | 238 | 17.00 |
| 66 | Medical comorbidities in autism: Challenges to diagnosis and treatment | Bauman, M. L. | NEUROTHERAPEUTICS | 2010 | 238 | 17.00 |
| 67 | Cerebrospinal fluid and serum markers of inflammation in autism | Zimmerman, A. W. | PEDIATRIC NEUROLOGY | 2005 | 238 | 12.53 |
| 68 | Elevated maternal C-reactive protein and autism in a national birth cohort | Brown, A. S. | MOLECULAR PSYCHIATRY | 2014 | 235 | 23.50 |
| 69 | Brain serotonin and dopamine transporter bindings in adults with high-functioning autism | Nakamura, K. | ARCHIVES OF GENERAL PSYCHIATRY | 2010 | 235 | 16.79 |
| 70 | Heart rate variability is associated with emotion recognition: Direct evidence for a relationship between the autonomic nervous system and social cognition | Quintana, D. S. | INTERNATIONAL JOURNAL OF PSYCHOPHYSIOLOGY | 2012 | 231 | 19.25 |
| 71 | Elevation of tumor necrosis factor-alpha in cerebrospinal fluid of autistic children | Chez, M. G. | PEDIATRIC NEUROLOGY | 2007 | 231 | 13.59 |
| 72 | Multisite functional connectivity MRI classification of autism: ABIDE results | Nielsen, J. A. | FRONTIERS IN HUMAN NEUROSCIENCE | 2013 | 230 | 20.91 |
| 73 | Evidence for a language quantitative trait locus on chromosome 7q in multiplex autism families | Alarcón, M. | AMERICAN JOURNAL OF HUMAN GENETICS | 2002 | 220 | 10.00 |
| 74 | Blood serotonin levels in autism spectrum disorder: A systematic review and meta-analysis | Gabriele, S. | EUROPEAN NEUROPSYCHOPHARMACOLOGY | 2014 | 216 | 21.60 |
| 75 | A failure of left temporal cortex to specialize for language is an early emerging and fundamental property of autism | Eyler, L. T. | BRAIN | 2012 | 216 | 18.00 |
| 76 | A genomewide scan identifies two novel loci involved in specific language impairment | Newbury, D. F. | AMERICAN JOURNAL OF HUMAN GENETICS | 2002 | 215 | 9.77 |
| 77 | Intranasal oxytocin treatment for social deficits and biomarkers of response in children with autism | Parker, K. J. | PROCEEDINGS OF THE NATIONAL ACADEMY OF SCIENCES OF THE UNITED STATES OF AMERICA | 2017 | 214 | 30.57 |
| 78 | A genomewide screen of 345 families for autism-susceptibility loci | Yonan, A. L. | AMERICAN JOURNAL OF HUMAN GENETICS | 2003 | 214 | 10.19 |
| 79 | Neuro-epileptic determinants of autism spectrum disorders in tuberous sclerosis complex | Bolton, P. F. | BRAIN | 2002 | 211 | 9.59 |
| 80 | Inflammation-related biomarkers in major psychiatric disorders: a cross-disorder assessment of reproducibility and specificity in 43 meta-analyses | Yuan, N. | TRANSLATIONAL PSYCHIATRY | 2019 | 210 | 42.00 |
| 81 | The possible role of the microbiota-gut-brain-axis in autism spectrum disorder | Srikantha, P. | INTERNATIONAL JOURNAL OF MOLECULAR SCIENCES | 2019 | 209 | 41.80 |
| 82 | Cytokine dysregulation in autism spectrum disorders (ASD): Possible role of the environment | Goines, P. E. | NEUROTOXICOLOGY AND TERATOLOGY | 2013 | 207 | 18.82 |
| 83 | Differences in fecal microbial metabolites and microbiota of children with autism spectrum disorders | Kang, D. W. | ANAEROBE | 2018 | 203 | 33.83 |
| 84 | Iron deficiency in pregnancy | Georgieff, M. K. | AMERICAN JOURNAL OF OBSTETRICS AND GYNECOLOGY | 2020 | 202 | 50.50 |
| 85 | Oxidative stress as an etiological factor and a potential treatment target of psychiatric disorders. Part 2. Depression, anxiety, schizophrenia and autism | Smaga, I. | PHARMACOLOGICAL REPORTS | 2015 | 202 | 22.44 |
| 86 | Review of neuroimaging in autism spectrum disorders: what have we learned and where we go from here | Anagnostou, E. | MOLECULAR AUTISM | 2011 | 202 | 15.54 |
| 87 | Autism and vitamin D | Cannell, J. J. | MEDICAL HYPOTHESES | 2008 | 202 | 12.63 |
| 88 | Transmission disequilibrium testing of arginine vasopressin receptor 1A (AVPR1A) polymorphisms in autism | Kim, S. J. | MOLECULAR PSYCHIATRY | 2002 | 202 | 9.18 |
| 89 | Associations between the oxytocin receptor gene (OXTR) and affect, loneliness and intelligence in normal subjects | Lucht, M. J. | PROGRESS IN NEURO-PSYCHOPHARMACOLOGY & BIOLOGICAL PSYCHIATRY | 2009 | 199 | 13.27 |
| 90 | Comparative genomics of autism and schizophrenia | Crespi, B. | PROCEEDINGS OF THE NATIONAL ACADEMY OF SCIENCES OF THE UNITED STATES OF AMERICA | 2010 | 196 | 14.00 |
| 91 | MEG detection of delayed auditory evoked responses in autism spectrum disorders: Towards an imaging biomarker for autism | Roberts, T. P. | AUTISM RESEARCH | 2010 | 193 | 13.79 |
| 92 | Functional connectivity classification of autism identifies highly predictive brain features but falls short of biomarker standards | Plitt, M. | NEUROIMAGE-CLINICAL | 2015 | 192 | 21.33 |
| 93 | Altered T cell responses in children with autism | Ashwood, P. | BRAIN BEHAVIOR AND IMMUNITY | 2011 | 192 | 14.77 |
| 94 | Glutathione redox imbalance in brain disorders | Gu, F. | CURRENT OPINION IN CLINICAL NUTRITION AND METABOLIC CARE | 2015 | 188 | 20.89 |
| 95 | Plasma cytokine profiles in subjects with high-functioning autism spectrum disorders | Suzuki, K. | PLOS ONE | 2011 | 184 | 14.15 |
| 96 | Atypical EEG complexity in autism spectrum conditions: A multiscale entropy analysis | Catarino, A. | CLINICAL NEUROPHYSIOLOGY | 2011 | 183 | 14.08 |
| 97 | Genomic screen and follow-up analysis for autistic disorder | Shao, Y. J. | AMERICAN JOURNAL OF MEDICAL GENETICS | 2002 | 183 | 8.32 |
| 98 | EEG analytics for early detection of autism spectrum disorder: A data-driven approach | Bosl, W. J. | SCIENTIFIC REPORTS | 2018 | 181 | 30.17 |
| 99 | Ketogenic diet modifies the gut microbiota in a murine model of autism spectrum disorder | Newell, C. | MOLECULAR AUTISM | 2016 | 181 | 22.63 |
| 100 | The many roads to mitochondrial dysfunction in neuroimmune and neuropsychiatric disorders | Morris, G. | BMC MEDICINE | 2015 | 180 | 20.00 |
